# Development of a Cyclic Voltammetric Method for the Determination of Cobalt(II) Ions Using o-Nitrosophenol

**DOI:** 10.1155/ianc/6675527

**Published:** 2025-09-19

**Authors:** Gulnora Karabayeva, Nigora Qutlimurotova, Zukhra Yakhshieva, Rukhiya Qutlimurotova, Nargiza Atakulova, Jasur Tursunqulov

**Affiliations:** ^1^Department of Analytical Chemistry, Jizzakh State Pedagogical University, Jizzakh, Uzbekistan; ^2^Department of Analytical Chemistry, National University of Uzbekistan, Tashkent, Uzbekistan; ^3^Department of Medical Biological Sciences, Kimyo International University, Tashkent, Uzbekistan; ^4^Department of Chemistry, Almalyk Branch of Tashkent State Technical University, Almalyk, Uzbekistan; ^5^Department of Pharmaceuticals and Chemistry, Alfraganus University, Tashkent, Uzbekistan

**Keywords:** acetate buffer, cobalt(II) ions, cyclic voltammetry, o*-*nitrosophenol, silver/mercury film working electrode

## Abstract

The demand for cobalt-based alloys has been steadily increasing due to advancements in industrial and cutting-edge technologies, particularly in metallurgy, where cobalt plays a crucial role in high-performance superalloys, battery production, and corrosion-resistant materials. Consequently, the concentration of cobalt ions in wastewater and environmental samples has exceeded permissible levels, raising significant ecological concerns. This study presents the development of an efficient method for the determination of cobalt(II) ions using a silver/mercury film working electrode (Hg(Ag)FE) modified with the organic dye *ortho-nitrosophenol* (o-NF) through cyclic voltammetry (CV). Optimization of the experimental conditions revealed that an acetate buffer (0.1 M, pH 5.1) served as the supporting electrolyte, with an accumulation time of 10 s and a concentration of 2.0 μM o-nitrosophenol. The preconcentration conditions were adjusted to enhance the sensitivity and selectivity for cobalt(II) ion detection. The method exhibited a linear relationship in the concentration range of 0.040–0.160 μM (*R*^2^ = 0.9863), with a limit of detection (LOD) of 0.010 μM and a limit of quantification (LOQ) of 0.034 μM for Co(II) ions. The proposed method was successfully applied to the analysis of water samples from the Aydar–Arnasoy Reservoir, and the accuracy of the results was statistically validated using Student's *t*-test. These findings demonstrate the potential of the developed method as an effective tool for environmental monitoring and the determination of cobalt ions in ecological protection initiatives.

## 1. Introduction

Cobalt(II), as a representative of heavy metals, accumulates in the environment—particularly in wastewater and soil—mainly as a result of industrial activities. Some of its compounds are environmentally stable and can exert toxic effects even at low concentrations [1]. Natural water bodies are considered one of the primary sources of cobalt in the biosphere for living organisms. Monitoring of the concentration of this element in natural waters is crucial for several reasons. Notably, cobalt is a structural component of vitamin B12 and acts as a specific activator of various enzymes. It occupies a central position in the vitamin B12 molecule, comprising 4.5% of its mass. Cobalt stimulates hematopoiesis, participates in the synthesis of heme from protoporphyrin and Fe, and prevents anemia [2].

The concentration of cobalt in the bloodstream ranges from 0.01 to 0.91 ng mL^−1^. Exceeding this range can lead to the development of various diseases [3]. The toxicity of cobalt and some of its compounds has been conclusively demonstrated [4], and in some cases, it exhibits carcinogenic effects [5]. Cobalt may contribute to the development of cancerous tumors and is included in the International Agency for Research on Cancer (IARC) list of carcinogens.

Special state policies and normative documents are being adopted to clean wastewater and soil contaminated with heavy metals and ensure their safe use. The need for highly efficient, cost-effective, and advanced analytical methods capable of detecting heavy metals at low concentrations is increasing to minimize environmental pollution and prevent the degradation of soil and water resources [6].

Currently various methods and techniques have been elaborated to detect heavy metals, particularly cobalt, which include flame atomic absorption spectroscopy (FAAS) [7], electrothermal atomic absorption spectrometry (ETAAS) [8], colorimetric detection method [9], gravimetric method [10], photometry [11], extended Raman spectroscopy [12], electrochemical method [13], atomic fluorescence spectrometry [14], laser ablation spectrometry [15], and inductively coupled plasma–mass spectrometry (ICP–MS) [16].

The analytical methods mentioned above, in addition to their high accuracy, require complex equipment, extended periods, or specialized laboratory conditions to perform the necessary measurements [17]. Recently considerable attention has been given to advanced electrochemical techniques for detecting heavy metals, including cobalt, due to their sensitivity, cost-effectiveness, and suitability for miniaturization [18–21]. Electrochemical methods are among the most effective techniques for detecting trace concentrations of essential metals in samples.  Table 1 presents a comparison of various electrochemical approaches for the determination of Co(II), including their detection limits, supporting electrolytes, and applied methodologies.

Studies conducted using various electrode types, complexing agents, and supporting electrolyte media have demonstrated the high sensitivity of Co(II) ion detection. In particular, nitrogen-containing organic ligands such as dimethylglyoxime (DMG), nioxime, and 1-nitroso-2-naphthol form stable complexes with cobalt, ensuring a wide linear range and low limits of detection (LODs). Supporting electrolytes, including ammonium, acetate, and borate buffer solutions, are commonly used; careful adjustment of their pH values plays a critical role in enhancing the electrochemical activity of Co(II) ions.

Among the electrodes, mercury-based types (hanging mercury drop electrode [HMDE], MFE, Hg(Ag)FE) and modified electrodes (e.g., Bi/MWNT–GCE, PbF–SPCE) stand out for their high sensitivity and low LOD values. Notably, the use of the silver amalgam film electrode (Hg(Ag)FE) significantly reduces the amount of toxic mercury required, thereby improving environmental safety. Importantly, the application of less commonly studied reagents such as nitrosophenol for cobalt ion detection enhances the innovative aspect of the method and presents a promising direction for future electroanalytical research.

The main objective of this study was to elaboratea method for the determination of cobalt(II) ions at nanomolar concentrations by forming a complex with the organic dye o-nitrosophenol. In this method, the electrochemical mechanisms and analytical procedures for detecting cobalt(II) ions are studied via a silver/mercury film working electrode (relative to Ag/AgCl) by cyclic voltammetry (CV). During this research, key parameters such as the total charge of the particles involved in the reaction near the electrode surface, the number of electrons, the half-wave potential, the influence of the background electrolyte, the pH value of the solution, and the composition of the complex were determined. These processes provide the optimal conditions for the highly sensitive detection of cobalt(II) ions.

The novelty of this study lies in the use of o-nitrosophenol, a rarely applied organic reagent in voltammetric methods, for the sensitive determination of Co(II) ions. The application of the silver/mercury film electrode (Hg(Ag)FE), which provides an environmentally safer alternative to conventional mercury electrodes, further enhances the practical value and sensitivity of the method.

## 2. Chemical Substances

o-Nitrosophenol (L) (“Almalyk MMC” JSC), acetic acid (70.0%), sodium hydroxide (99.0%), cobalt(II) nitrate hexahydrate (99.95%), nitric acid (99.95%), metallic mercury (P-00), and potassium chloride (99.0%) were used in the experimental process. All other reagents and chemicals employed met the analytical grade standards. The pH values of the solutions were adjusted by adding the required amounts of acetic acid or sodium hydroxide.

## 3. Experimental Part

For the CV experiments, voltammograms were taken in three-electrode electrochemical cells using a solid silver/mercury film working electrode (Hg(Ag)FE), a saturated Ag/AgCl reference electrode (saturated with 1 M KCl), and a Pt wire counter electrode. A 0.01 M solution of crystalline Co(NO_3_)_2_ × 6H_2_O (99.95%) containing Co^2+^ ions was prepared by dissolving 0.291 g of salt in a 100 mL volumetric flask, which was filled with distilled water, and then titrated complexometrically with EDTA [37]. Further solutions were prepared by diluting this mixture. A 0.1 M acetate buffer solution was prepared by adding 1 N 167.4 mL acetic acid and 50 mL 1 N sodium hydroxide to the mark in a 500 mL volumetric flask, which was filled with distilled water.

CV curves were recorded in a 0.1 M acetate buffer (pH 5.1) containing 2 μM o-nitrosophenol. The total volume of the examined solution was 25 mL, and all measurements were carried out at room temperature. In this experiment, a deposition potential of 50 mV was initially applied, followed by a potential range of 40–500 mV at a scan rate of 5–11 mV/s. The accumulation time for cobalt ions on the electrode surface was 10 s. The three electrodes were immersed in the solution, and the ion distribution was made uniform by stirring the solution at 400 rpm (revolutions per minute) via a magnetic stirrer. To remove dissolved oxygen, nitrogen gas was bubbled through the solution for 15 min. After these procedures, the solution was allowed to settle, and CV measurements were recorded by applying a potential scan rate of 5–11 mV/s. The procedure and schematic for preparing the mercury layer of the silver amalgam electrode are described in the article [38]. The electrolysis process was conducted after 10 measurement cycles to refresh the solution, ensuring satisfactory reproducibility of the results.

## 4. Results and Discussion

### 4.1. Effects of pH and Buffer Volume

**Figure 1:**
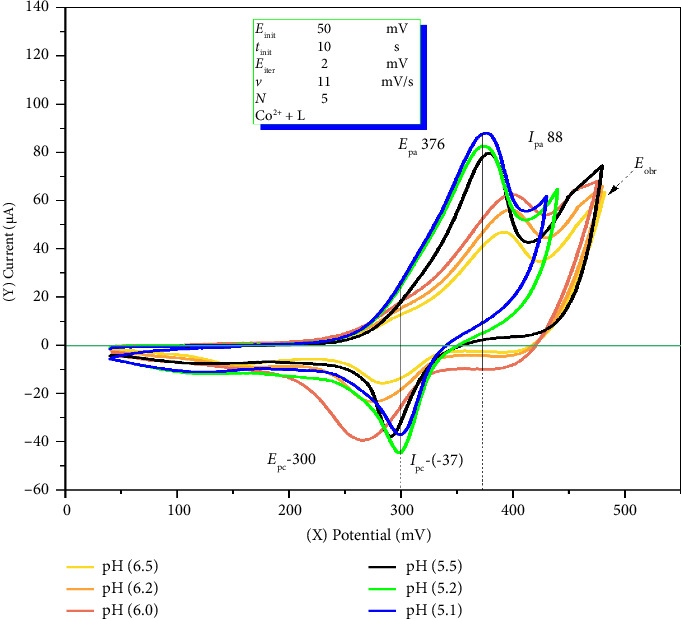
Influence of pH on the complexation of cobalt ions under cyclic voltammetry conditions accumulation time (τ_a_cc): 10 s, preconcentration potential 50 mV). Experimental parameters: Co^2+^ ion concentration of 0.040 μM, o-nitrosophenol concentration of 2.0 μM, with a metal-to-ligand molar ratio of 1:50, and an acetate buffer as the supporting electrolyte, with pH values of 5.1, 5.2, 5.5, 6.0, 6.2, and 6.5.

**Figure 2:**
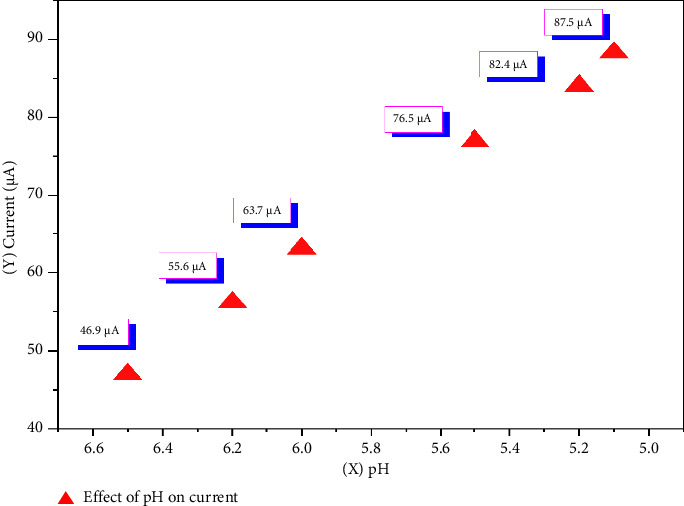
Effect of pH on the peak current (*I*_*p*_) under cyclic voltammetry conditions.

The pH and concentration of the acetate buffer play a crucial role in the complexation of Co^2+^ ions with nitroso-containing compounds. Notably, within the pH range of 5.0–5.5, the buffer solution stabilizes complex formation and significantly enhances the electrochemical response [35]. To further investigate this effect, CV measurements were performed using buffer solutions within the pH range 5.0–6.5. The results, shown in Figures 1 and 2, clearly illustrate the relationship between buffer pH, complex stability, and electrochemical response optimization.

The optimal pH range of 5.0–5.5 was found to favor the formation of the Co(II)–o-nitrosophenol (Co^2+^–o-NF) complex in a slightly acidic medium. A decrease in the analytical signal was observed when the background electrolyte pH was varied from 5.0 to 6.5. Notably at pH 5.1 a higher anodic current response was recorded (Figure 2), indicating efficient protonation of Co^2+^ ions, which facilitates the continuous progression of the complex formation reaction with o-NF. In the pH range 6.0–6.5, a decline in the analytical signal was observed, which can be attributed to the precipitation of Co^2+^ ions, preventing complete complexation. Furthermore, the analytical signal at pH 5.1 was found to be higher than that at pH 5.5. Therefore, to optimize the kinetics of complex formation between Co^2+^ ions and the analytical reagent, as well as to ensure reliable electrochemical analysis, an acetate buffer with a pH of 5.1 and an accumulation time of 10 s was selected as the optimal condition.

**Figure 3:**
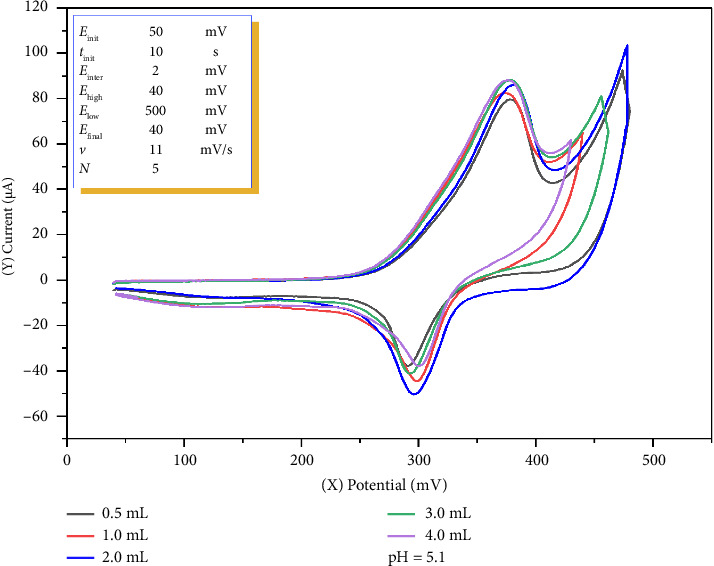
Effect of acetate buffer volume (0.1 M, pH 5.1) on the anodic and cathodic peak currents of the Co^2+^–o-NF complex in cyclic voltammetry.

To further evaluate the effect of the supporting electrolyte, the influence of acetate buffer volume on the electrochemical behavior of the Co^2+^–o-NF complex was investigated. Cyclic voltammetric measurements were carried out at pH 5.1 using varying buffer volumes of 0.5, 1.0, 2.0, 3.0, and 4.0 mL. The results are summarized in Figure 3.

The experimental data showed that at 0.5 and 1.0 mL of buffer, both anodic and cathodic peak currents were relatively low. When 2.0 mL of buffer was used, a noticeable increase in the anodic peak current was observed, and the cathodic peak current reached its maximum value. At buffer volumes of 3.0 and 4.0 mL, the anodic peak remained high, but the peak current values were almost identical, overlapping each other in the voltammograms. However, under these conditions the cathodic peak currents were considerably lower than those obtained with 2.0 mL of buffer.

Based on these findings, 2.0 mL of 0.10 M acetate buffer was identified as the optimal condition. This volume provided a strong and stable anodic response, also yielding the highest cathodic peak current, making it the most favorable condition for the electroanalytical determination of the Co^2+^–o-NF complex.

### 4.2. Calculation of the Diffusion Coefficient of o-Nitrosophenol

**Figure 4:**
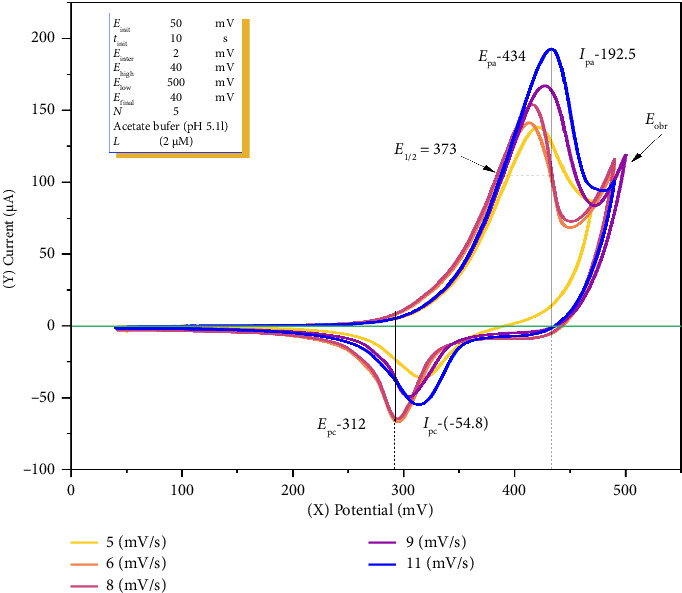
Cyclic voltammogram of ortho-nitrosophenol.

To calculate the diffusion coefficient of o-NF via CV, a solution of 30 mL total volume was prepared in a quartz glass electrochemical cell consisting of 2 μM o-NF, 2.0 mL of 0.1 M acetate buffer (pH 5.1), and 22.95 mL of distilled water. In the 12–20 mV/s scan rate range, it was not possible to measure the anodic and cathodic peaks, as the analytical signal appeared as a broadened curve. Therefore, the maximum current was obtained within the 5–11 mV/s potential range (Figure 4, Table 2).

Measurements were conducted with an accumulation time of 10 s, a preconcentration potential of 50 mV, a potential scan rate of 5–11 mV/s, and a potential range of 40–500 mV, using 2.0 μM o-NF in a 0.1 M acetate buffer solution at pH 5.1.

The half-wave potential (*E*_1/2_ = 0.374 V) was calculated at the optimal scan rate of 0.011 V/s using Formula (1) where the anodic peak potential (*E*_pa_) was 0.434 V and the cathodic peak potential (*E*_pc_) was 0.314 V:

(1) $$\begin{array}{c} E_{1/2}=\frac{E_{\text{pa}}+E_{\text{pc}}}{2}, \end{array}$$

where *E*_pa_ is the anodic peak potential, *E*_pc_ is the cathodic peak potential. *E*_1/2_ values presented in the table were determined using this equation [39].

The Randles–Sevcik equation (2) is one of the most effective and widely utilized methods for predicting the maximum current in electrochemical processes. This equation highlights that the square root of the diffusion coefficient is directly proportional to the analytical signal, with the molecular mass and solvent composition playing significant roles in the relationship. Furthermore, the surface area of the electrode can substantially increase the magnitude of the analytical signal. On the basis of the experimental data obtained, the diffusion coefficients for both the anodic and the cathodic current intensities of o-nitrosophenol were determined via the Randles–Sevcik equation [40–42]. The computed values are presented in Table 3.

(2) $$\begin{array}{c} I_{p}=\left(2.69\times 10^{5}\right)n^{3/2}A\;C\;D^{1/2}v^{1/2}. \end{array}$$

Based on the experimental data, the half-wave potential for the oxidation and reduction of o-nitrosophenol at the working electrode was determined to be 0.374 V. The anodic peaks were observed within the range 0.414–0.434 V, while the cathodic peaks appeared in the range 0.294–0.320 V (at a scan rate of 5–11 mV/s). The anodic current intensities ranged from 138 to 192 μA, exhibiting positive values, whereas the cathodic current intensities varied from –37.0 μA to –55.0 μA, displaying negative values. The observed difference in diffusion coefficients between the anodic and cathodic processes suggests that the oxidation of o-nitrosophenol at the working electrode occurs at a higher rate than its reduction. This finding supports the electrochemical redox process of the nitroso (-NO) group, which can be represented by the following reaction equations:

#### 4.2.1. Anodic Process (Oxidation)

In the anodic process, o-nitrosophenol (Ar–NO) is oxidized to o-nitrophenol (Ar–NO_2_):

(3) $$\begin{array}{c} \text{Ar}–\text{NO}+H_{2}O⟶\text{Ar}–NO_{2}+2H^{+}+2e \end{array}$$

#### 4.2.2. Cathodic Process (Reduction)

In the cathodic process, o-nitrophenol (Ar–NO_2_) is reduced back to o-nitrosophenol (Ar–NO) or further to hydroxylamine derivative (Ar–NHOH), depending on the number of electrons and protons involved:

(4) $$\begin{array}{c} \text{Ar}–NO_{2}+2H^{+}+2e\;⟶\text{ Ar}–\text{NO}{+H}_{2}O, \end{array}$$

or

(5) $$\begin{array}{c} \text{Ar}–\text{NO}{+2H}^{+}{+2e}^{-}⟶\text{Ar}–\text{NHOH}. \end{array}$$

These electrochemical transformations confirm the redox behavior of o-nitrosophenol in CV, highlighting its ability to undergo oxidation to o-nitrophenol and reduction back to either the nitroso or hydroxylamine state, depending on the experimental conditions [43].

The observed Δ*E*_*p*_ values exceed the theoretical reversible limit of 59 mV for a one-electron transfer process, indicating that the redox process of o-nitrosophenol involves a two-electron transfer mechanism. The larger Δ*E*_*p*_ suggests a quasi-reversible or irreversible electrochemical behavior, implying that the reduction and oxidation processes do not occur with ideal reversibility. This can be attributed to slow electron transfer kinetics or additional chemical steps coupled to the redox reaction, which hinder the rapid re-equilibration of the redox species at the electrode surface. Consequently, the redox process of o-nitrosophenol is more challenging in comparison with a simple one-electron transfer reaction.

The electrochemical parameters of cobalt ions, including their half-wave potential and diffusion coefficient, were determined and compared with those of o-nitrosophenol. The study examined the changes in the diffusion coefficient of cobalt ions, the shift in half-wave potential, and the effect of scan rate on cyclic voltammograms (CVs). The obtained results are presented in the appendix (Appendix Figure 9).

### 4.3. Effect of the Scan Rate on the Formation of Complexes Between Cobalt Ions and o-Nitrosophenol

The scan rate plays a crucial role in the sensitivity and accuracy of the analysis. The variation in the potential scan rate demonstrated a linear increase in the anodic peak current within the 5–11 mV/s scan rate range. Anodic peak current (*I*_pa_) and scan rate (ν) exhibited a linear trend in the investigated range (5–11 mV/s), indicating that the electron transfer process is primarily diffusion-controlled. However, at higher scan rates (≥ 9 mV/s), the increase in *I*_pa_ slows down, suggesting the presence of adsorption effects or kinetic limitations at the electrode surface. The peak potential difference (Δ*E*_*p*_) increases with the scan rate, indicating that the electron transfer kinetics are partially quasi-reversible. Additional electrochemical techniques (e.g., chronoamperometry) could be employed to further evaluate mass transport and kinetic parameters.

The maximum analytical signal (*I*_*p*_), under constant temperature conditions, is dependent on the constants *R* and *F*, and the number of electrons (*n*) and the diffusion coefficient (*D*) in the oxidation–reduction process of the formed complex remain constant. However, the values of these parameters may vary depending on factors such as the scan rate, concentration, and electrode composition in CV experiments. Therefore, to obtain a more thorough and complete analysis of the complex, voltammograms obtained under optimal conditions should be utilized. The effect of the potential scan rate on the electrochemical catalytic reaction of Co^2+^ ions with o-NF was investigated via CV. After a 10-s accumulation time and preconcentration potential of 50 mV, the results were obtained in the 5–11 mV/s scan rate range.

**Figure 5:**
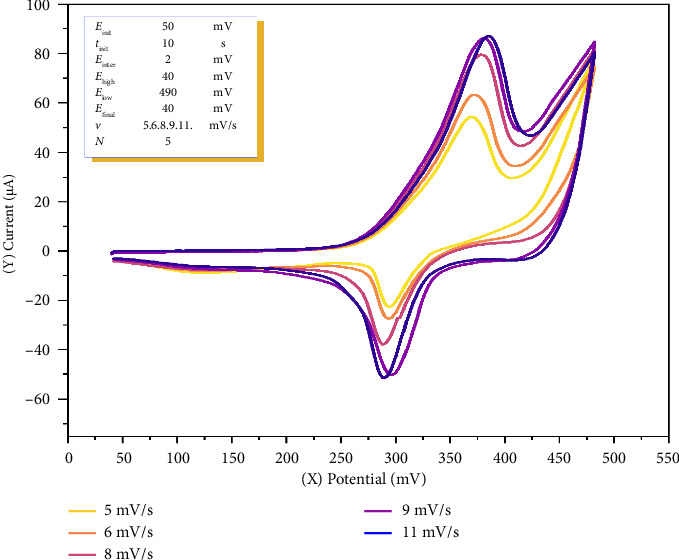
Voltammograms showing the effect of the potential scan rate on the electrochemical behavior of the system with a 10-s accumulation time in an acetate buffer solution (pH 5.1). Measurements were conducted using an Hg(Ag)FE (*A* = 0.277 cm^2^) at scan rates of 5, 6, 8, 9, and 11 mV/s. The solution contained 0.040 μM Co^2+^ and 2.0 μM o-nitrosophenol.

To determine the diffusion coefficient of cobalt(II) ions with o-NF via CV, a 30 mL quartz glass cell was prepared containing 0.040 μM Co^2+^, 2.0 μM o-NF, 0.1 M 2.0 mL acetate buffer at pH 5.1, and 22.94 mL of distilled water, resulting in the formation of a total of 25 mL of solution. The voltammograms of the electrochemical complex formed between cobalt(II) ions and o-NF were recorded at a potential scan rate of 5–11 mV/s (Figure 5, Table 4).

As shown in Figure 4, the increase in potential led to an increase in both the anodic and cathodic peak currents. This phenomenon is attributed to the higher scan rate reducing the diffusion layer and enhancing the faradaic current. In the analysis of a metal ion, the maximum current is determined via deconvolution, and this method is not used for the analysis of other compounds [44]. According to the deconvolution results, the oxidized and reduced peaks of the cobalt ion were identified.

There is a clear correlation between the scan rate and potential, where an increase in the scan rate shifts the potential to a more positive value. Moreover, to facilitate the oxidation reaction, an increase in the scan rate necessitates an increase in potential. In this case, the optimal measurement condition for the Co^2+^–o-NF solution was found to be 11 mV/s, as this scan rate provided the highest analytical signal, which is likely due to favorable electrochemical kinetics at this rate. Further analysis was conducted at this scan rate.

At scan rates higher than 11 mV/s, a decrease in current was observed, indicating a non-ohmic nature. According to Ohm's law, an increase in potential should lead to an increase in current, but in this process, owing to the typical electrochemical nature, the current decreases due to the nonfaradaic or capacitive zone.

On the basis of the voltammogram values presented in Table 5, the diffusion coefficients of the anodic and cathodic currents in the complex formation of cobalt(II) with o-nitrosophenol were calculated according to Equation (2), similar to those for o-nitrosophenol. The obtained results are presented in Table 5.

Based on the obtained data, the formation of a complex between cobalt(II) and o-nitrosophenol at the working electrode results in a half-wave potential of 0.337 V, with anodic peaks observed in the range of 0.370–0.384 V and cathodic peaks within 0.288–0.296 V, at a scan rate of 5–11 mV/s. The anodic current ranged from 54.0 to 87.0 μA, while the cathodic current varied from −22.0 to −51.0 μA. The potential differences between the anodic and cathodic peaks for the Co^2+^–o-NF complex, relative to o-nitrosophenol alone, were 0.044–0.050 V and 0.006–0.024 V, respectively. Furthermore, the differences in anodic and cathodic current values—ranging from 88.0 to 105.0 μA and from 15.0 to 16.0 μA, respectively—indicate a direct correlation with complex formation. The observed differences in half-wave potentials and diffusion coefficients between o-nitrosophenol and the Co^2+^–o-NF complex further support complex formation. Additionally, the higher cathodic diffusion coefficient of the cobalt-nitrosophenol complex in comparison with to o-nitrosophenol suggests that the reformation of Co^2+^ ions occurs owing to dissociation of the complex.

### 4.4. Calculation of the Number of Electrons Involved in the Formation of the Cobalt(II)–o-Nitrosophenol Complex

**Figure 6:**
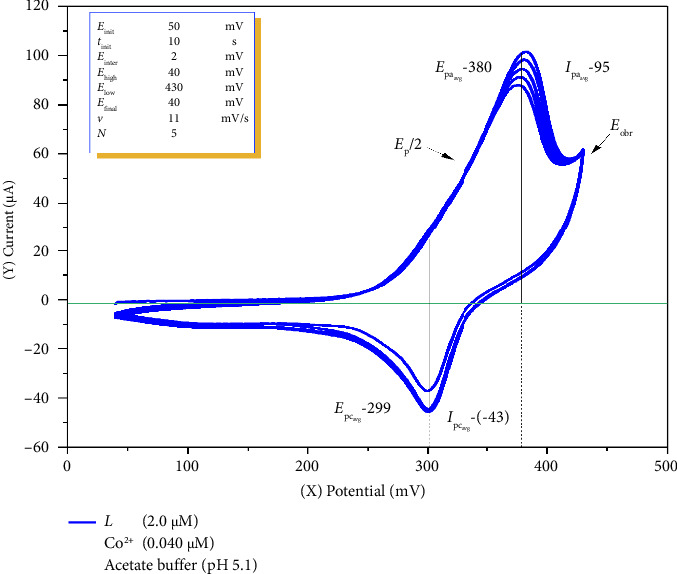
Voltammetric response of cobalt ions with o-nitrosophenol at the working electrode at a scan rate of 11 mV/s (2.0 μM o-NF, 0.040 μM Co^2+^, acetate buffer at pH 5.1, accumulation time 10 s, preconcentration potential 50 mV, and potential range from 40 to 500 mV).

The catalytic activity of the Co(II)–o-nitrosophenol complex produced maximum anodic and cathodic current intensities at a scan rate of 11 mV/s. Therefore, the number of electrons involved in this process was calculated via CV at this scan rate on the basis of the optimal conditions selected above. Parallel determinations were performed five times (Figure 6). On the basis of the voltammetric values, the number of electrons involved in the formation of the cobalt–nitrosophenol complex was calculated, and a reaction mechanism was proposed accordingly.

**Scheme 1:**
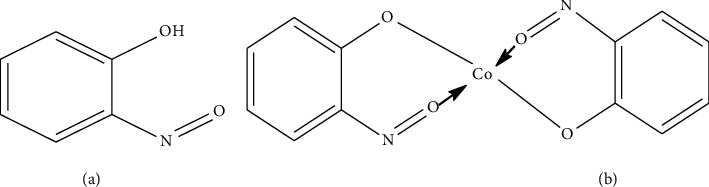
(a) Molecular structure formula of o-nitrosophenol. (b) Chemical structure formula of the complex formed by o-nitrosophenol with Co(II) ions.

In CV, the primary role of diffusion is to ensure separation between the anodic (oxidation) and cathodic (reduction) peaks. For *n* = 1 electron, the maximum separation is approximately 59 mV, and for other values of *n*, this separation is given by 59/*n*. The presence of background substances in the electrolyte, as well as the solvent and other impurities, can modify these separation values, causing them to shift either to the right or left. These shifts necessitate accounting for the influence of interfering species when analyzing electrochemical processes and reactions. On the basis of the values presented in the voltammogram results on Figure 6, the following calculations were performed.

(6) $$\begin{array}{c} n=\frac{I_{\text{pa}}}{I_{\text{pc}}/1.106},\\ \left(\text{ratio of }1.106-\text{ideal values}\right), \end{array}$$

and

(7) $$\begin{array}{c} n=\frac{0.059}{{\Delta E}_{p}},\\ \left(\text{Laviron equation}\right), \end{array}$$

for the oxidation–reduction pair of the complex formed by o-NF with Co(II). It was calculated that *n* = 2 according to the Laviron equations [40, 45], and on this basis, the reaction mechanism was expressed as follows:

Based on the experimental data obtained from CV measurements, the oxidation process of o-NF in the supporting electrolyte was identified to occur at 0.434 V. This anodic peak potential (*E*_pa_) corresponds to the redox activity of the free molecular form of o-NF (Scheme 1(a)) and represents its oxidation to nitrophenol (Equation (3)). This value reflects the behavior of o-NF when it has undergone redox transformation independently at the electrode surface.

Upon addition of Co^2+^ ions into the solution, a coordination complex is formed on the electrode surface between o-NF molecules and cobalt(II) ions (Scheme 1(b)), represented by the following equilibrium reaction:

Complex formation reaction (anodic reaction):

(8) $$\begin{array}{c} 2\;o-\text{NO}-C_{6}H_{4}-\text{OH}+{\text{Co}}^{2+}⇌\;\left[\text{Co}{\left(o-\text{NO}-C_{6}H_{4}-O\right)}_{2}\right]+2H^{+}. \end{array}$$

Electrochemical investigation of the resulting complex revealed a shift of the anodic peak potential (*E*_pa_) to 0.384 V. This shift of the anodic potential to a lower value (compared to the free ligand) indicates that the oxidation process of o-NF becomes thermodynamically more favorable upon complex formation. Such a shift arises from changes in electron density at the coordination, the binding of the ligand to the metal center, and stabilization effects within the complex.

In the subsequent cathodic process, the o-NF moiety coordinated within the complex is electrochemically reduced to its hydroxylamine derivative in a protic (aqueous acetate) medium, consistent with data reported in the literature [46, 47]. This reduction peak is observed at 0.290 V, which is slightly lower than the *E*_pc_ value of the free o-NF (*E*_pc_ 0.314 V), indicating that the redox transformation occurs in the coordinated state. Following the reduction, the complex dissociates, resulting in the formation of the hydroxylamine product at the electrode surface and the release of cobalt ions back into the solution as free Co^2+^ species. This process can be represented by the following reaction:

Reduction of coordinated o-nitrosophenol to hydroxylamine (cathodic reaction):

(9) $$\begin{array}{c} \left[\text{Co}{\left(o-\text{NO}-C_{6}H_{4}-O_{2}\right)}_{2}\right]+2H^{+}⟶{\text{Co}}^{2+}+2o-\text{NO}-C_{6}H_{4}-\text{OH} \end{array}$$

(10) $$\begin{array}{c} o-\text{NO}-C_{6}H_{4}-\text{OH}+{2H}^{+}+{2e}^{-}⟶\text{HO}-\text{NH}-C_{6}H_{4}-\text{OH} \end{array}$$

The electrochemical half-reactions presented above confirm that the redox process of the cobalt(II)–o-nitrosophenol complex involves a two-electron exchange mechanism. The oxidation reaction shows the loss of two electrons, while the reduction reaction demonstrates their gain, ensuring charge balance within the system. The voltammetric data, particularly the peak potential separation (Δ*E*_*p*_) and the application of the Laviron equation, further validate the *n* = 2 electron transfer process. This consistency with theoretical electrochemical principles supports the proposed reaction mechanism.

Further experimental validation, including spectroelectrochemical studies or additional electrochemical modeling, could provide deeper insights into the reaction mechanism.

A critical point to note is that the observed redox processes do not involve the oxidation of cobalt from Co^2+^ to its higher oxidation state, Co^3+^. According to literature data, the oxidation potential of Co^2+^ to Co^3+^ lies in the negative potential range (typically below −0.2 V) [48], which is far from the potential window observed in this study (0.337–0.374 V). Therefore, the redox changes observed in the voltammograms are exclusively related to o-nitrosophenol and its coordinated form, with no contribution from cobalt oxidation.

### 4.5. Influence of the Cobalt Ion Concentration on the Analytical Signal

**Figure 7:**
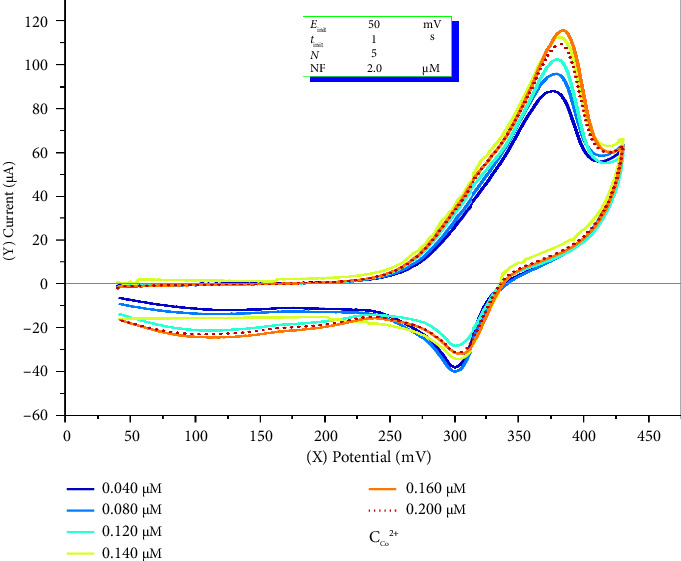
Effect of the cobalt(II) ion concentration on the analytical signal, as illustrated by the voltammogram.

**Figure 8:**
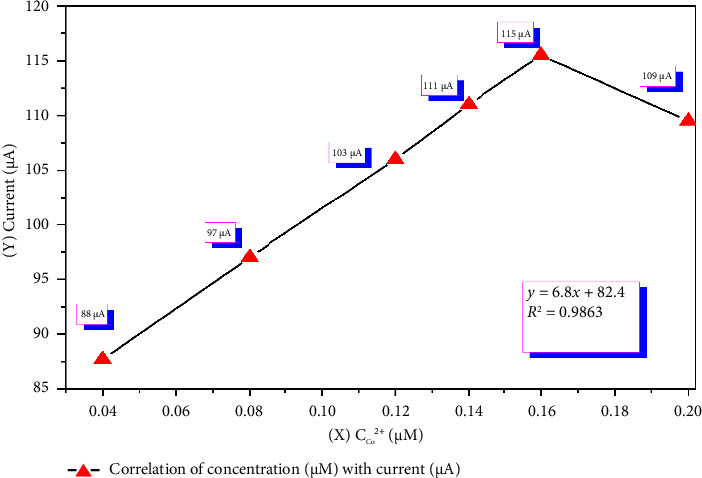
Linear relationship between the cobalt(II) ion concentration and the analytical signal.

The effect of the cobalt ion concentration on the CV curve was meticulously analyzed under predetermined optimal conditions. The experiments were conducted after a 10-s accumulation time, within a potential range of 40–500 mV, at a potential scan rate of 11 mV/s, and an acetate buffer with a pH of 5.1 was used. A total solution volume of 25 mL was prepared, containing 2.0 μM o-NF and varying cobalt ion concentrations of 0.040, 0.080, 0.120, 0.140, 0.160, and 0.200 μM. The results obtained under these conditions are illustrated in Figures 7 and 8.

On the basis of the CV results presented in Figure 8, a linear increase in the analytical signal was observed as the concentration of Co^2+^ ions increased within the range of 0.040–0.200 μM, whereas deviations occurred at concentrations exceeding this range. This phenomenon is explained by the complex correlation laws between the molecular mass and diffusion coefficient (*D*) in different solvents [49]. Initially, the increase in concentration is associated with an increase in number of charge carriers in the solution and their enhanced mobility, leading to an increase in the current. However, beyond a certain concentration, an excess of charge carriers results in a buildup in their movement pathways, which in turn leads to a decrease in diffusion rates. As a consequence, a reduction in the analytical signal is observed. The primary factors affecting electrical conductivity include the mobility of ions and electrons, as well as the internal structural interactions of the substances involved, which govern the dynamics of charge carrier motion.

To validate the accuracy of the developed method, errors were determined via the “inserted-determined” approach on the basis of the voltammogram values obtained from CV for the cobalt–nitrosophenol complex. The correlation coefficient between the analytical signal and the concentration of cobalt(II) ions in the range 0.040–0.160 μM was calculated as *R* = 0.9863, indicating the high accuracy and reliability of the developed method Figure 8, Table 6.

Based on the obtained results (Sr, *S*, and *R*^2^), the LOD and the limit of quantification (LOQ) for cobalt(II) ions using the developed cyclic voltammetric method were determined to be 0.010 and 0.034 μM, respectively. A strong linear relationship was observed in the concentration range 0.040–0.160 μM, with a correlation coefficient (*R*) = 0.9863.

### 4.6. Influence of Interfering Ions on the Voltammetric Detection of Cobalt(II) Ions

To assess the selectivity of the developed method, the influence of interfering ions was systematically investigated. For this purpose a 30 mL quartz cell was filled with 2.0 μM of o-nitrosophenol, 2.0 mL of 0.1 M acetate buffer at pH = 5.1, and 0.170 μM Co(II) with metal ions at ratios of 1:0.5 Ni(II), 1:0.41 Fe(II), 1:0.12 Cu(II), 1:0.45 V(III), 1:0.27 Ba(II), 1:0.32 Fe(III), 1:0.19 Pb(II), 1:0.18 Mn(II), and 1:0.40 Cd(II). The total volume was adjusted to 25 mL by adding distilled water. Voltammetric data were obtained by applying a scanning potential of 11 mV/s to the electrode. The results were calculated via the “insert-find” method and are presented in Table 7.

On the basis of the obtained data, the voltammetric determination of 0.170 μM cobalt(II) ions did not reveal interference at the ratios indicated in the table. However, in natural systems, the concentrations of foreign ions may be significantly higher or lower than the ratios provided. In particular, when nickel(II), copper(II), cadmium(II), iron(II), and lead(II) ions are present at higher concentrations than cobalt(II), they negatively affect the voltammetric determination of cobalt(II). In such cases the use of masking reagents for these ions can eliminate their interference, enhancing the accuracy of the analysis. These masking reagents are listed in Table 8.

These reagents block the interference of foreign ions by forming complexes, thereby preventing them from negatively affecting the determination of cobalt(II) ions.

### 4.7. Comparative Evaluation of the Proposed Method

In order to assess the scientific novelty of the obtained research results and to ensure their reliability and accuracy, a comparative analysis was conducted against existing literature on the voltammetric determination of cobalt. For this purpose, the analytical parameters selected for this study were systematically compared with previously reported voltammetric methods for cobalt determination, as summarized in Table 9.

The results presented in Table 9 highlight the key advantages and limitations of the proposed method compared to existing voltammetric techniques for cobalt determination.

#### 4.7.1. Advantages

The silver–mercury film electrode is more environmentally friendly and stable compared to the traditional HMDE.

The use of acetate buffer (pH 5.1) allows analysis under near-neutral acidic conditions, broadening its application potential.

The complexing agent o-nitrosophenol represents a novel approach that has not been widely explored in previous studies.

The short accumulation time (τ_a_cc = 10 s) offers a significant practical advantage, enabling rapid analysis.

This method has been successfully applied for the first time to analyze cobalt in the Aydar–Arnasoy water reservoir.

#### 4.7.2. Limitations and Future Improvements

The LOD is higher than that of some highly sensitive techniques, indicating the need for further sensitivity enhancement.

The linear range is relatively narrow and could be expanded in future studies to improve its applicability across a broader concentration range.

### 4.8. Validation and Application of the Developed Method

In binary, ternary, and more complex mixtures simulating real-world environments and industrial materials, the relative standard deviation for the cyclic voltammetric determination of cobalt(II) ions did not exceed 0.33. This demonstrates the potential applicability of the developed method for analyzing environmental objects and materials of various natures (natural and waste water, concentrates, ores, minerals, and other relevant materials). Furthermore, the method was successfully applied for the determination of Co^2+^ ions in the Aydar–Arnosoy Reservoir. To assess the accuracy of the method, the results were compared with those obtained from inductively coupled plasma optical emission spectrometry (Avio200 ISP–AES, Perkin Elmer, USA), and the results are presented in Table 10.

The determination of Co(II) ions in Aydar–Arnosoy Reservoir water was carried out via the CV method as follows. Initially, 2 mL of 0.1 M acetate buffer (pH = 5.1), 2.0 μM o-nitrosophenol solution, and 23 mL of water from the Aydar–Arnosoy Reservoir were added to a quartz cuvette. After allowing the solution to settle, CVs were recorded. The results are presented in Table 11.

According to the table results, the calculated Student coefficient tp_cal_ = 2.750 is smaller than the tabulated value tp_tab_ = 2.776. Therefore, tp_tab_ > tp_cal_, which indicates that the calculated value is smaller than the tabulated value, confirming the accuracy of the elaborated method and suggesting the absence of systematic errors.

## 5. Conclusion

The optimal conditions for the CV method for the determination of cobalt(II) ions using o-nitrosophenol were developed. A high analytical signal and maximum complex formation were observed in a weakly acidic acetate buffer solution. The anodic diffusion coefficient of o-nitrosophenol (*D*_ox_ = 1.83 × 10^−5^ cm^2^/s) was higher than the cathodic diffusion coefficient (*D*_re_ = 2.33 × 10^−6^ cm^2^/s), indicating a stronger oxidation process of the nitroso group. Upon complexation with cobalt(II) ions, the anodic diffusion coefficient (*D*_ox_ = 5.48 × 10^−6^ cm^2^/s) decreased significantly, which can be attributed to reduced mobility of the complex and the involvement of functional groups. In contrast, the cathodic diffusion coefficient of the complex (*D*_re_ = 9.79 × 10^−6^ cm^2^/s) exceeded that of the free reagent, indicating complex decomposition and enhanced mobility due to the release of nitroso, hydroxyl, and cobalt ions. The half-wave potential of free o-nitrosophenol was found to be 0.374 V, while that of its cobalt(II) complex shifted negatively to 0.337 V, confirming interaction with the metal ions. The number of electrons involved in the redox process was determined to be two, confirming a 1:2 stoichiometric ratio between cobalt(II) and the ligand. The analytical performance of the proposed method was evaluated using both graphical and regression-based approaches. The methods exhibited a linear relationship in the concentration range of 0.040–0.160 μM (*R*^2^ = 0.9863), with a LOD of 0.010 μM and a LOQ of 0.034 μM for Co(II) ions. These findings demonstrate the method's high sensitivity and reliability for cobalt(II) detection. Its selectivity can be enhanced using masking agents. Successful application to Aydar–Arnasoy Reservoir' water samples and validation via Student's *t*-test confirm its suitability for environmental analysis.

In future studies, the method may be extended to the simultaneous determination of multiple metal ions in complex matrices such as industrial wastewater and biological fluids. Furthermore, modifying the electrode surface with nanomaterials or ion-imprinted polymers could further enhance the method's selectivity and sensitivity.

## Figures and Tables

**Figure 9:**
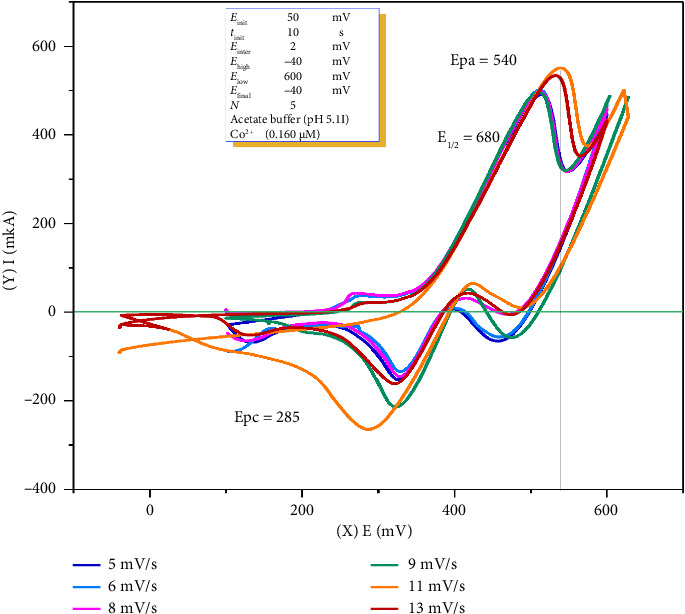
Cyclic voltammograms of cobalt ions at different scan rates.

**Table 1:** Comparison of electrochemical methods for cobalt(II) detection.

No.	Electrode material	Supporting electrolyte composition	Complexing reagent for Co(II)	Linear range	Limit of detection (LOD)	Accumulation time (τ_a_cc s)	Analyzed matrices
[22]	Polyacrylic acid–modified glassy carbon electrode (PAA/GCE)	Aacetate buffer pH 2.0–11.0	Polyacrylic acid (PAA)	0.125–2.0 mM	11.0 µM	300 s	Tap vater
[23]	Bismuth film-modified graphite screen-printed electrode (Bi–GSPE)	0.1 M ammonia/ammonium buffer solution pH 9.0	Dimethylglyoxime (DMG)	10–60 μg/L	2.4 μg/L	120 s	Soil and battery recycling samples
[24]	Antifouling gel–integrated microelectrode array	Borate buffer, pH 8.5	Nioxime	—	0.27–0.29 nM	90 s	Freshwater, seawater
[25]	Pt working electrode	Buffer at pH 7.0	—	510–723 mg/L	217.06 mg/L	—	Wastewater sample
[26]	Hanging mercury drop electrode (HMDE)	0.1 M acetate buffer, pH 5.1	1-Nitroso-2-naphthol (1N2N)	0.1–5.0 mg/L	0.03 mg/L	—	Aqueous solution
[27]	Bi/MWNT-modified glassy carbon electrode	0.01 mol/L ammonia buffer solution pH 9.0	Dimethylglyoxime (DMG)	4.0 × 10^−10^–1.0 × 10^−7^ mol/L	8.1 × 10^−11^ mol/L	200 s	Seawater, lake water
[28]	Lead film screen-printed carbon electrode (PbF–SPCE)	0.2 M ammonia buffer pH 8.7	1,2-Cyclohexanedione dioxime (nioxime)	0.03–0.59 mg/L	0.003 mg/L	120 s	Aqueous samples
[29]	In situ Pb film on carbon fiber microelectrode (3-electrode microset)	0.2 M NH_4_OH/H_2_SO_4_ pH 7.8–9.5	Nioxime (1,2-cyclohexanedione dioxime)	2 × 10^−10^–1 × 10^−8^ mol/L	9.5 × 10^−11^ mol/L	240 s	Certified reference water (SPS–SW1)
[30]	Mercury film electrode (MFE)	0.02 M NH_3_/NH_4_Cl buffer, 2 × 10^−4^ M DMG	Dimethylglyoxime (DMG)	0.15–0.5 mg/kg	0.15 mg/kg	10 s	Soil and airborne particulate matter
[31]	Hanging mercury drop electrode (HMDE)	0.02 M NH_3_/NH_4_Cl buffer, pH 9.0	2-Aminocyclopentene-1-dithiocarboxylic acid (ACDA)	0.10–40 ng/mL	0.1 ng/mL	60 s	Water, blood plasma
[32]	Mercury film electrode (MFE)	0.1 M acetate buffer, pH 4.8	4-(2-pyridylazo) resorcinol (PAR)	0.5–20 μg/L	0.1 μg/L	120 s	Tap water, river water
[33]	Rotating disk mercury-film electrode (MFE)	—	Dimethylglyoxime (DMG)	0.08–2.40 μg/dm^3^	0.016 μg/dm^3^	60 s	Aqueous solution
[33]	Mercury film electrode (MFE)	0.1 M ammonia buffer, pH 9.2	2,3-Dimercaptopropane-1-sulfonate (DMPS)	0.02–5.0 μg/L	0.005 μg/L	120 s	River water, tap water
[34]	Silver amalgam film electrode (Hg(Ag)FE)	0.1 M ammonia buffer, pH 9.2	Cyclohexanedione dioxime (Nioxime)	1.7 × 10^−10^–1.2 × 10^−7^ M	5.8 × 10^−11^ M (0.0035 μg/L)	60 s	Rainwater samples
[35]	Carbon paste electrode (CPE)	0.1 M acetate buffer, pH 5.0	Nitroso-S	3.3–187.1 μg/L	1.8 μg/L	—	Drinking water, natural water
[36]	UV–Vis spectrophotometer	Acetate buffer, pH 4.5	1-Nitroso-2-naphthol (1N2N)	0.1–5.0 mg/L	0.03 mg/L	—	Water samples

**Table 2:** Voltammetric parameters of o-nitrosophenol at different potential scan rates (5–11 mV/s, *N* = 5).

Scan rate (V/s)	*E*_pa_ (V)	*E*_pc_ (V)	Δ*E*_p_ (V)	*E*_1/2_ (V)	*I*_pa_ (μA)	*I*_pc_ (μA)
0.005	0.422	0.320	0.102	0.371	138.0	−37.0
0.006	0.414	0.294	0.120	0.354	141.0	−67.0
0.008	0.416	0.294	0.122	0.355	153.0	−65.0
0.009	0.428	0.304	0.124	0.366	167.0	−50.0
0.011	0.434	0.314	0.120	0.374	192.0	−55.0

**Table 3:** Diffusion coefficients of o-nitrosophenol.

No.	*v* (V/s)	*I*_pa_ (μA)	*I*_pc_ (μA)	D_anodic (cm^2^/s)	D_cathodic (cm^2^/s)
1	0.005	138.0	−37.0	2.11 × 10^−5^	1.52 × 10^−5^
2	0.006	141.0	−67.0	1.84 × 10^−5^	4.15 × 10^−6^
3	0.008	153.0	−65.0	1.62 × 10^−5^	2.93 × 10^−6^
4	0.009	167.0	−50.0	1.72 × 10^−5^	1.54 × 10^−6^
5	0.011	192.0	−55.0	1.86 × 10^−5^	1.53 × 10^−6^
Avg				1.83 × 10^−5^	2.33 × 10^−6^

*Note:* (*T* = 293.15 K, *A* = 0.277 cm^2^, *n* = 2, C_o−NP_ = 2.0 μM).

**Table 4:** Voltammetric parameters of Co^2+^ and o-nitrosophenol at different potential scan rates (5–11 mV/s, *N* = 5).

Scan rate (V/s)	*E*_pa_ (V)	*E*_pc_ (V)	Δ*E*_*p*_ (V)	*E*_1/2_ (V)	*I*_pa_ (μA)	*I*_pc_ (μA)
0.0050	0.370	0.294	0.076	0.332	54.0	−22.0
0.0060	0.374	0.292	0.082	0.333	63.0	−26.0
0.0080	0.378	0.288	0.090	0.333	80.0	−38.0
0.0090	0.382	0.296	0.086	0.339	86.0	−50.0
0.0011	0.384	0.290	0.094	0.337	87.0	−51.0

*Note:* The half-wave potential *E*_1/2_ = 0.337 V.

**Table 5:** Diffusion coefficients of Co^2+^ and o-nitrosophenol.

No.	*v* (V/s)	*I*_pa_ (μA)	*I*_pc_ (μA)	D_anodic (cm^2^/s)	D_cathodic (cm^2^/s)
1	0.005	54.0	−22.0	8.10 × 10^−6^	3.15 × 10^−6^
2	0.006	63.0	−26.0	9.17 × 10^−6^	9.67 × 10^−6^
3	0.008	79.0	−38.0	1.08 × 10^−5^	7.19 × 10^−6^
4	0.009	86.0	−50.0	1.14 × 10^−5^	3.64 × 10^−6^
5	0.011	87.0	−51.0	9.50 × 10^−6^	3.78 × 10^−6^
Avg				9.79 × 10^−6^	5.48 × 10^−6^

*Note:* (*T* = 293.15 K, *A* = 0.277 cm^2^, *n* = 2, C_Co_ = 0.040 μM).

**Table 6:** Evaluation of the accuracy and precision of the cyclic voltammetry method for detecting cobalt(II) ions with o-nitrosophenol.

Entered C_Co_^2+^ (μM)	Current (μA)	Found μM $\overset{-}{X}$ ± Δ*X*	*S*	Sr
0.040	87.70	0.041 ± 0.0012	0.0009	0.023
0.080	95.80	0.079 ± 0.0012	0.0009	0.012
0.120	102.5	0.118 ± 0.0012	0.0009	0.009
0.140	111.5	0.410 ± 0.0013	0.0011	0.003
0.160	115.8	0.158 ± 0.0013	0.0010	0.006
0.200	109.5	0.198 ± 0.0013	0.0010	0.005

**Table 7:** Effect of interfering cations on the accuracy and reproducibility of the voltammetric determination of cobalt(II) ion.

Foreign cation	Entered C_x_ μM	C_Co_^2+^/C_x_	Found C_Co_^2+^ μM; ($\overset{¯}{x}$ ±Δ*X*; *p* = 0.95)	*N*	*S*	Sr
Nickel(II)	0.340	0.50	0.1695 ± 0.0006	5	0.00051	0.003
Iron(II)	0.424	0.41	0.1689 ± 0.0012	5	0.00100	0.006
Copper(II)	1.420	0.12	0.1683 ± 0,0020	5	0.00153	0.009
Vanadium(III)	0.380	0.45	0.1688 ± 0.0014	5	0.00119	0.007
Barium(II)	0.630	0.27	0.1688 ± 0.0014	5	0.00119	0.007
Iron(III)	0.530	0.32	0.1686 ± 0.0027	5	0.00238	0.014
Lead(II)	0.890	0.19	0.1671 ± 0.0030	5	0.00238	0.014
Manganese(II)	0.940	0.18	0.1676 ± 0.0029	5	0.00255	0.015
Cadmium(II)	0.420	0.40	0.1650 ± 0,0053	5	0.00460	0.028

*Note:* (C_Co_^2+^ = 0.170 μM; τ_a_cc = 10 s; *E*_pre_ = 50 mV; *E*_1/2_ = 0.337 V).

**Table 8:** Reagents blocking Ni(II), Cu(II), Cd(II), Fe(II), and Pb(II) ions in the voltammetric determination of cobalt(II) (pH = 5.0–6.0).

Destructive ions	Blocking reagents	Optimal concentration
Nickel(II)	EDTA	0.05 M
Copper(II)	Tartaric acid	0.05 M
Cadmium(II)	DTPA	0.05 M
Iron(II)	Ascorbic acid	0.05 M
Lead(II)	Thiosulfate	0.05 M

**Table 9:** Comparative analysis of analytical parameters for the voltammetric determination of cobalt obtained in this study and reported in the literature.

No.	Authors	Method	Electrode material	Supporting electrolyte	Complexing agent	Catalytic additive	Linear range	Limit of detection (LOD)	Accumulation time	Analyzed matrix
[50]	Refera et al. (1998)	DPASV	Modified CPE	Sodium acetate (pH 6)	N-p-Chlorophenylcinnamohydroxamic acid (CPCHA)	None	1 × 10^−6^–4 × 10^−5^ M	3.3 × 10^−7^ M	5 min	Vitamin B12
[51]	Sancho (2000)	DPCSV	HMDE	NH_3_ (pH 9.2)	Dimethylglyoxime	None	1–100 μg/kg	1.1 μg/kg	60 s	Beet sugar solutions
[52]	Ellwood and Van Den Berg (2001)	CSV	HMDE	NH_3_-NH_4_Cl (pH 9.1); NaNO_2_	Nioxime	None	10–103 pM	∼3 pM	60–120 s	Atlantic seawater
[53]	Korolczuk and Tyszczuk (2006)	AdSV (Pb FE)	Lead-film electrode	NH_3_ (pH 8.2), NaNO_2_	Nioxime	NaNO_2_	0.1–5 nM	0.041 nM	120 s	Certified water sample
[54]	B. Rezaei and E. Rezaei (2006)	AdCSV	HMDE	NH_3_/NH_4_Cl (pH 8.9)	Dimethylglyoxime	None	1–1000 μg/L	0.8 μg/L	Not mentioned	Galvanic wastewater
[55]	Ardakani et al. (2008)	CV (ZME)	Zeolite-modified CPE	NaNO_3_, phosphate buffer	Zeolites (A,X,Y)	None	3–10 ppm	3 ppm	15 min	Water samples
[56]	Tyszczuk et al. (2009)	AdSV (Pb FE)	Lead-film electrode	PIPES (pH 6.8), CTAB	Nioxime	CTAB	5 × 10^−10^–2 × 10^−8^ M	1.1 × 10^−11^ M	120–600 s	Rainwater sample
[57]	Bobrowski et al. (2013)	SW–CAdSV	Hg(Ag)FE	NH_3_ (pH 9.2), NaBrO_3_	Dimethylglyoxime	NaBrO_3_	0.5–100 nM	0.1 nM	60 s	Rainwater sample
[58]	Alves et al. (2013)	SW–AdCSV	Bi–VE	NH_3_ (pH 9.2)	Dimethylglyoxime	None	0.5–16 μg/L	0.09 μg/L	30–90 s	River water
[59]	Rutyna and Korolczuk (2015)	AdSV	Lead-film electrode	NH_3_, NaNO_2_	Nioxime	NaNO_2_	1.18–58.9 ng/L	0.47 ng/L	120–300 s	Seawater (NASS-5)
[60]	Deswati et al. (2015)	AdSV	HMDE	NH_4_Cl (pH 7)	Calcon	None	0.2–120 μg/L	0.232 μg/L	70 s	Sea, drinking, river water
[61]	Torkashvand et al. (2016)	CSDPV (IIP)	IIP/magnetic nanoparticles–GCE	NH_3_ buffer (pH 9)	8-Hydroxyquinoline	None	0.5–500 nM	0.1 nM	120 s	Water, serum, urine
[62]	This study	CV	Hg(Ag)FE	Acetate buffer (pH 5.1)	o-Nitrosophenol	None	0.040–0.160 µM	0.010 µM	10 s	Aydar–Arnasoy water reservoir

**Table 10:** Elemental analysis results of Co^2+^ ions and other elements in the Aydar–Arnosoy Reservoir composition based on ISP–AES analysis (С [μM]).

Element	Mg	Cr	As	Li	K	Ca	Fe	Co	Ni	Cu
С (μM)	0.1650	0.1347	0.0267	72.94	35.24	652.58	0.179	0.1697	0.1533	0.0157

**Element**	**Zn**	**Al**	**B**	**Se**	**Sn**	**Sb**	**Pb**	**V**	**Mo**	**Cd**

С (μM)	0.0459	2.631	202.47	0.6712	3.75	0.7805	0.2075	1.236	0.6774	0.0267

**Table 11:** Results of applying the developed method to the Aydar–Arnasoy water analysis.

Method	C_Co_^2+^ in water quantity μM	Found di C_Co_^2+^ μM; ($\overset{¯}{x}$ ± Δ*X*; *p* = 0.95)	*N*	*S*	Sr	*t*_*p*_ table	*t*_*p*_ calculated
CV	0.1697	0.1544 ± 0.016	5	0.013	0.084	2.776	2.750

*Note:* (C_Co_^2+^ = 0.1697 μM; τ_a_cc = 10 s; *E*_1/2_ = 0.337 V, N = 5, *p* = 0.95).

## Data Availability

The original data and materials associated with this study are included within the article. Further inquiries regarding the data can be directed to the corresponding author(s).
